# Structural and Functional Analyses of Five Conserved Positively Charged Residues in the L1 and N-Terminal DNA Binding Motifs of Archaeal RadA Protein

**DOI:** 10.1371/journal.pone.0000858

**Published:** 2007-09-12

**Authors:** Li-Tzu Chen, Tzu-Ping Ko, Yu-Wei Chang, Kuei-An Lin, Andrew H.-J. Wang, Ting-Fang Wang

**Affiliations:** 1 Institute of Biochemical Sciences, National Taiwan University, Taipei, Taiwan; 2 Department of Life Sciences, National Taiwan University, Taipei, Taiwan; 3 Institute of Biological Chemistry, Academia Sinica, Taipei, Taiwan; 4 National Core Facility of High-Throughput Protein Crystallography, Academia Sinica, Taipei, Taiwan; Vanderbilt University, United States of America

## Abstract

RecA family proteins engage in an ATP-dependent DNA strand exchange reaction that includes a ssDNA nucleoprotein helical filament and a homologous dsDNA sequence. In spite of more than 20 years of efforts, the molecular mechanism of homology pairing and strand exchange is still not fully understood. Here we report a crystal structure of *Sulfolobus solfataricus* RadA overwound right-handed filament with three monomers per helical pitch. This structure reveals conformational details of the first ssDNA binding disordered loop (denoted L1 motif) and the dsDNA binding N-terminal domain (NTD). L1 and NTD together form an outwardly open palm structure on the outer surface of the helical filament. Inside this palm structure, five conserved basic amino acid residues (K27, K60, R117, R223 and R229) surround a 25 Å pocket that is wide enough to accommodate anionic ssDNA, dsDNA or both. Biochemical analyses demonstrate that these five positively charged residues are essential for DNA binding and for RadA-catalyzed D-loop formation. We suggest that the overwound right-handed RadA filament represents a functional conformation in the homology search and pairing reaction. A new structural model is proposed for the homologous interactions between a RadA-ssDNA nucleoprotein filament and its dsDNA target.

## Introduction

The RecA family of DNA strand exchange proteins exists in all three kingdoms of life. Members of the RecA protein family include prokaryotic RecA, archaeal RadA and Rad51, and eukaryotic Rad51 and Dmc1. These proteins play a central role in homologous recombination, an error-free DNA repair mechanism [for reviews see 1], [2], [3]. While RecA-deficient *E. coli* cells or Rad51-deficient yeast cells are viable, Rad51-deficient vertebrate cells are not. The latter accumulate chromosomal breaks prior to cell death [4]. Rad51 and its meiosis-specific homolog, Dmc1, are indispensable for meiosis [5], [6]. Mammalian Rad51 and Dmc1 proteins interact with tumor suppressor proteins, such as BRCA2 [7], [8]. Together, RecA family proteins have important roles in cell proliferation, genome maintenance, and genetic diversity, particularly in higher eukaryotes.

Most of our understanding of the mechanism of RecA family proteins comes from *E. coli* studies. In all current models, RecA molecules load onto ssDNA, forming a *6_1_* right-handed helical filament with six monomers per helical turn in the presence of ATP and scan for homologous dsDNA. Once homology is recognized, a synaptic complex, consisting of a three-stranded DNA filament, forms. Eventually, the DNA strands are exchanged and ATP is hydrolyzed, resulting in the displacement of one of the original duplex strands, and the creation of a new heteroduplex (or D-loop) is created. The molecular mechanisms underlying these processes are not fully understood.

Members of RecA family all contain a central ATPase domain that is preceded by a short β-stranded polymerization motif (PM). The central ATPase domain not only mediates a classical ATP-induced allosteric effect that causes large-scale changes in protomers and filament assembly, it also contains two disordered loops (denoted L1 and L2 motifs) for ssDNA binding [9]. The PM is responsible for the assembly of helical filaments and toroidal rings [9]–[16]. In RadA, Rad51, and Dmc1, a conserved phenylalanine residue in the PM docks into a hydrophobic pocket on the neighboring core ATPase domain. A similar interaction was also observed with a fusion protein containing the human Rad51 monomer and a peptide from the BRCA2 protein [10], [17]. Immediately following the PM and before the central ATPase domain, we recently identified a highly flexible region, called subunit rotation motif (SRM). Clockwise rotation of the SRM along the axes of RadA protein polymers is responsible for quaternary structural transitions from a protein ring to a *6_1_* right-handed filament with six monomers per helical turn, then to a *3_1_* overwound right-handed filament with three monomers per helical turn and finally to a *4_3_* left-handed filament with four monomers per helical turn [see Figure 5 in 16]. RadA, Rad51 or Dmc1 each has an additional N-terminal domain (NTD) that interacts with dsDNA [18], [19], and RecA has a small C-terminal domain (CTD), for which a similar function has been proposed. The helix-hairpin-helix (HhH) motif in the NTD mediates dsDNA binding [18], [19].

The biochemistry of the ATP-dependent strand exchange reaction was recently reviewed in detail [20]. In some models, RecA family proteins function as DNA pairing enzymes, and ATP hydrolysis facilitates RecA dissociation and/or distribution along DNA substrates. These models do not explain the bypass of non-homologous DNA sequences during the homology pairing and strand exchange reactions. Alternatively, the facilitated DNA rotation model proposes that RecA family proteins function as motor proteins that coordinate rotation between dsDNA and ssDNA. The facilitated DNA rotation model seemed compatible with all experimental results obtained to date [20]. Despite some mechanistic differences, all current models of RecA family proteins share the principle that these proteins function as *6_1_* right-handed helical filaments throughout their catalytic cycles, including the homology pairing and strand exchange reactions. This creates a problem for the facilitated DNA rotation model. Since all known DNA binding motifs (i.e., L1, L2, NTD, CTD) are localized along or near the central axes of *6_1_* right-handed helical filaments [9], [ 12]–[14], [see Figure 3D in 16], [21], the facilitated DNA rotation model implies that novel DNA binding sites are located on the exterior of the right-handed helical filaments to facilitate DNA rotation [20]. To date, such novel DNA binding sites have not been identified in any RecA family protein, and the mechanisms underlying protein-DNA and ssDNA-dsDNA interactions are not clear.

Recent structural studies indicate that large structural variations of RecA family protein filaments can occur. For example, X-ray crystallographic analyses revealed that *Sulfolobus solfataricus* RadA (*Sso*RadA) proteins could self-polymerize into a *3_1_* overwound right-handed filament [this study, 15] and a *4_3_* left-handed filament [16]. Using atomic force microscopy (AFM) with carbon nanotube tips, we showed that *Sso*RadA [see Figure 3B in ref. 22] and budding yeast *S. cerevisiae* Dmc1 [see supporting data Figure S1 in ref. 16] could each form both right- and left-handed helical filaments in solution. In addition, EM imaging analysis also revealed that RadA protein could form filaments with both right- and left-handed helical pitches [16]. These results suggest that left-handed filaments exist not only in protein crystallization conditions but also in neutral pH solutions. Moreover, the ability to form left-handed helical filaments is likely a general property of most RecA family proteins.

**Figure 1:**
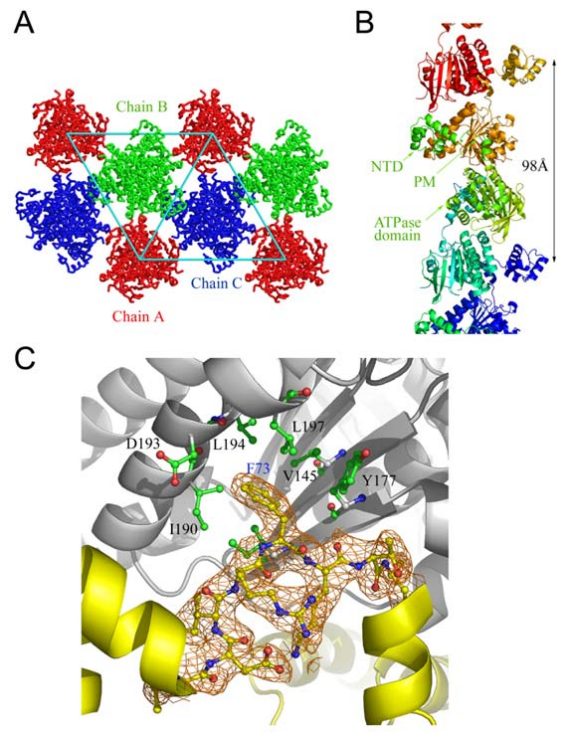
Crystal packing and quaternary structures. (A) *Sso*RadA protomers packed into three extended helical filaments. Chain A was located at the origin of the unit cell, whereas chains B and C were located one-third and two-third diagonal to the unit cell. (B) Side view of the *Sso*RadA right-handed helical filament crystal structure. The helical pitch of the filament is 98 Å. Each protomer is shown in a different color. The N-terminal domain (NTD), polymerization motif (PM), and central ATPase domain are indicated. (C) The Phe73 of the PM is buried in the hydrophobic pocket of the neighboring ATPase domain. Several hydrophobic residues that interact with the Phe73 side chain are indicated. The interactions result in the assembly of *Sso*RadA protomers into a filament. 2*F*_o_–*F*_c_ electron density maps (contoured at 1.0 σ), corresponding to the PM are shown in orange.

It is noteworthy that other investigators might have noted left-handed RecA family protein filaments. Yu and Egelman first reported in 1990 that *E. coli* RecA filaments formed on linear dsDNA in the presence of ATP and aluminum fluoride showed both right- and left-handed helical pitches [23]. Aluminum fluoride is able to substitute for phosphate after the hydrolysis of ATP, and it was used to trap the ADP-Pi state of the RecA protein. However, after the report of the crystal structure of *E. coli* RecA *6_1_* right-handed protein filament [9], this filament form became generally accepted as the active form of RecA protein. The left-handed helical filaments were then judged to be experimental artifacts due to the negative staining EM protocols. Specifically, it was postulated that visual impression of the left-handed filaments might result from deformation of flexible filaments during the absorption to EM grids. Such deformations may break the axial symmetry and cause the superposition of the signals from the upper and lower part of the filament, thus giving a visual impression of inclined striation. Although this interpretation seems reasonable, it also has pitfalls. First, it arose from a preconception that RecA and RecA family proteins could only form helical filaments with right-handed pitches. Second, to our knowledge, it was not verified by other experimental approaches, e.g., AFM or X-ray crystallography. As described above, we have recently reported the crystal structure of *Sso*RadA left-handed helical filament. In addition, using the AFM imaging approach, we [16], [22] and others (see below) have observed left-handed helical filaments of various RecA family proteins. For example, the cover picture of the May 1, 1998 issue of *Genes and Development* shows an AFM image of a left-handed helical filament made by the *Sso*RadA proteins; however, the paper described only right-handed filaments [24]. It would be interesting to know if the image was inadvertently mirrored. Moreover, another recent AFM imaging study revealed a single RecA-dsDNA helical filament with both right- and left-handed helical pitches [Figure 1E in 25]. These results together, raise the possibility that the *6_1_* right-handed helical filaments may not be the only functional conformation for RecA family proteins.

In this study, we present the crystal structure of *Sso*RadA *3_1_* overwound right-handed filament at 1.93Å resolution. Although this structure is similar to that reported previously [15], it reveals unprecedented geometric/conformational details in the L1 motif and the NTD due to its higher resolution. Subsequent biochemical analyses indicate that five conserved basic amino acid residues (K27, K60, R217, R227, R229) in the L1 motif and the NTD are involved in DNA binding and in promoting formation of D-loop between a ssDNA and its complementary strand in the dsDNA target. We describe these results and propose a new structural model for DNA binding and homologous pairing.

## Results

### Overall Structure

In the final 1.93Å resolution crystal structure, *Sso*RadA protomers are packed into three extended helical filaments in a 98Å pitch helix with three protomers per turn (Figure 1A and 1B). Chain A is located at the origin of the unit cell, whereas chains B and C are located one-third and two-thirds diagonal to the unit cell, respectively (Figure 1A). Chains B and C run in opposite directions. This arrangement is similar to the recently reported *P3_1_21* form at 3.2 Å resolution [15]. Chains B and C have average B-values of 38.6 Å^2^ and 38.1 Å^2^, respectively. In contrast, chain A does not contain a visible NTD and has an average B-value of 48.2 Å^2^ (Table 1). A portion of chain A filaments may run in the opposite direction or has partial occupancy.

**Table 1:** Data collection and refinement statistics for the RadA crystal.

Data Types	Variables	Number
Collection	Space group	*P3_1_*
	Unit cell *a*, *b, c* (Å)	99.55, 99.55, 99.41
	Resolution (Å)	30–1.93 (2.00–1.93)
	Number of observations	260120 (18570)
	Unique reflections	81660 (7557)
	Completeness (%)	98.6 (91.4)
	Average I/σ(I)	20.2 (1.9)
	R_merge_ (%)	5.4 (44.9)
Refinement	Number of reflections	78018 (6168)
	R_work_ (95% data)	0.237 (0.346)
	R_free _(5% data)	0.295 (0.345)
	R_work_ (twin)	0.191 (0.306)
	R_free _(twin)	0.241 (0.320)
	R.m.s.d bond distance (Å)	0.018
	R.m.s.d bond angle (deg)	1.7
	Ramachandran plot (% non-Gly & non-Pro residues)	
	In most favored regions	92.6
	In additional allowed regions	7.4
	Average B (Å^2^)/No. of non-H atoms	
	A-chain protein	48.2/1799
	B-chain protein	38.6/2254
	C-chain protein	38.1/2245
	Water molecules	54.1/1099

All positive reflections were used in the refinement. Numbers in parentheses are for the highest resolution shell.

As in other filamentous structures of archaeal and eukaryotic RecA family proteins [12]–[16], a conserved amino acid residue (Phe73) in the PM is responsible for the assembly of the *3_1_* overwound right-handed *Sso*RadA filament. Phe73 is docked into a hydrophobic pocket (which includes Val145, Tyr177, Ile190, Asp193, Leu194, and Leu197) of the neighboring protomer (Figure 1C). Intriguingly, the overwound right-handed *Sso*RadA filament contains ordered polypeptide backbones and side-chains in two important structural regions, L1 motif (amino acid residues 219–226) and NTD (amino acid residues 14–69), compared to those found in the previous lower resolution *P3_1_21* form. These new structural details allow better modelling of protein-DNA and ssDNA-dsDNA interactions.

### Structure of the L1 motif

**Figure 2:**
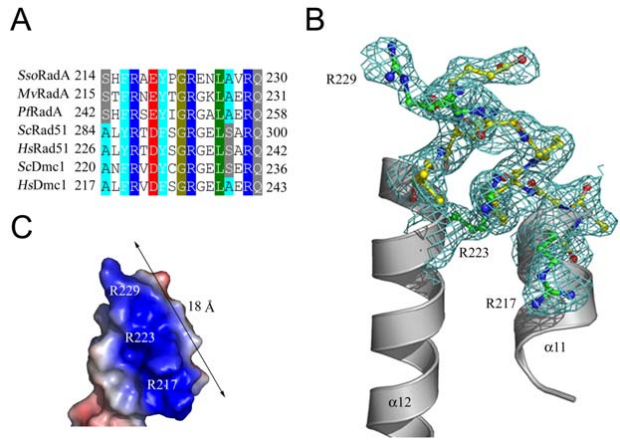
Structure of the L1 motif. (A) Sequence alignment of RadA homologs from *S. solfataricus* (*Sso)* RadA, *M. voltae* (*Mv*) RadA, *P. furiosus* (*Pf*) Rad51, *H. sapiens* (*Hs)* Rad51 and Dmc1, and *S. cerevisiae* (*Sc*) Rad51 and Dmc1. Three conserved arginine residues are shown in cyan. (B) A ribbon diagram of the L1 motif showing two alpha-helices (grey). The hinge region is depicted with a ball-and-stick model (yellow). The side chains of three conserved arginine residues are shown in green. 2*F*_o_–*F*_c_ electron density maps (contoured at 1.0σ), corresponding to the ssDNA binging site, are shown in cyan. (C) Surface charge potential of the L1 motif. The positively and negatively charged regions are indicated in blue and red, respectively. The linear basic patch is ∼18 Å in length.

A previous structure of the *Methanococcus voltae* RadA (*Mv*RadA) *6_1_* right-handed helical filament at 2.4 Å resolution indicated that L1 and L2 form positively charged patches compatible for binding anionic DNA [21]. In our *Sso*RadA *3_1_* overwound right-handed helical filament, the L1 motif (Figure 2A) is located between the α11 and α12 helices (Figure 2B). A surface charge potential analysis of the L1 motif reveals a linear basic patch on one face of the motif (Figure 2C). This linear basic patch of *Sso*RadA L1 motif consists of three arginine residues (i.e., Arg217, Arg223, and Arg229), with their positively charged side chains directed upward. Intriguingly, Arg217, Arg223, and Arg229 are conserved in all archaeal and eukaryotic RecA family proteins (Figure 2A) but not in *E. coli* RecA protein. This difference may explain the previous observation that human Rad51 and *E. coli* RecA exhibit profound mechanistic differences in ssDNA binding [26].

### Structure of the NTD

**Figure 3 pone-0000858-g003:**
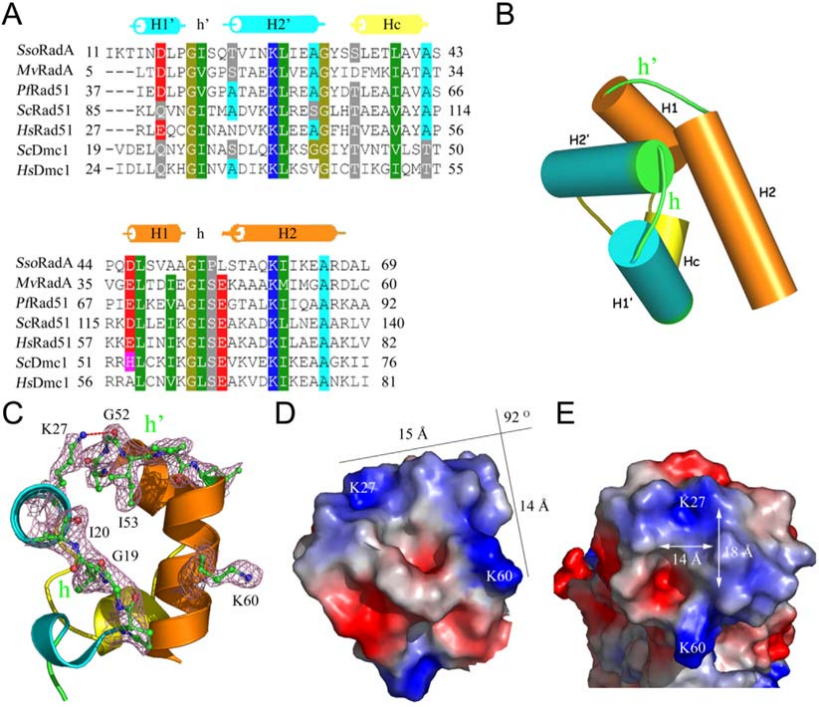
Structure of the N-terminal domain (NTD). (A) Sequence alignment of the NTD and (HhH)_2_ domains in RadA homologs from *S. solfataricus*(*Sso*) RadA), *M. voltae* (*Mv*) RadA, *P. furiosus* (*Pf*) Rad51, *H. sapiens*(*Hs*) Rad51 and Dmc1, and *S. cerevisiae* (*Sc*) Rad51 and Dmc1. The first and second HhH motifs are denoted as H1'h'H2' and H1hH2, respectively. (B) Cartoon diagram of the (HhH)_2_ domains. The first and second HhH motifs are connected by the connector alpha-helix Hc (yellow). (C) A ribbon diagram of the NTD. The hinge regions (h' and h) are depicted with a ball-and-stick model (green). 2*F*_o_–*F*_c_ electron density maps (contoured at 1.0 σ) of several key amino acid residues are in purple. Oxygen and nitrogen atoms are shown in red and blue, respectively. (D) Surface charge potential of the NTD. The positively and negatively charged regions are indicated by blue and red, respectively. The two borders of this 92° arched basic patch are 15 Å and 14 Å in length, respectively. (E) Top view of the NTD surface charge potential reveals a central channel that is 18 Å long and 14 Å wide. The channel is too narrow to accommodate a B-type dsDNA substrate.

The NTD is actually an (HhH)_2_ domain, in which a pseudo two-fold unit is composed of two HhH motifs linked by an α-helix [27]. The HhH motifs and the connector α-helix are denoted as H1'h'H2', H1hH2, and Hc (Figure 3A). Each HhH motif contains two helices (denoted as H1, H1', H2, or H2') and a hairpin (denoted as h or h') (Figure 3B). Alignment of the archaeal RadA and eukaryotic Dmc1 and Rad51 protein sequences clearly shows the conservation of a GΦ pattern in the two hairpins, where G is glycine and Φ is a hydrophobic residue (e.g., Ile, Val, or Leu) (Figure 3A). The GΦ pattern completes the hydrophobic core within each HhH motif. In contrast, the connector helix, Hc, links the two HhH motifs and completes the hydrophobic core of the entire (HhH)_2_ domain (Figure 3C).

Previous studies of other enzymes involved in DNA metabolism (e.g., rat polymerase β, *E. coli* AlkA base excision repair glycosylase, and human 8-oxoguanine DNA glycolysase) revealed that their (HhH)_2_ domains are capable of inducing dsDNA bending and led to distortion and/or flipping of base pairs [28]–[30]. Surface potential analysis of the entire (HhH)_2_ domain of *Sso*RadA protein revealed a 92° arched basic patch along the border of the second HhH motif (Figure 3D). Two lysine residues (i.e., Lys27 and Lys60) are located at each end of this arched basic patch. Lys27 is part of the first HhH motif, and its amino group contacts the carbonyl group of Gly52 (Figure 3C). In contrast, Gly52 and Lys60 are part of the second HhH motif. Importantly, the Lys27, Gly52, and Lys60 residues are conserved in other archaeal RadA proteins and eukaryotic Rad51 and Dmc1 proteins (Figure 3A). The corresponding residues in human Rad51 are Lys40, Gly65, and Lys73. A previous nuclear magnetic resonance study revealed that Gly65 and Lys73 of human Rad51 are directly involved in dsDNA binding [18]. Conceivably, RadA, Rad51, and Dmc1 may utilize this arched basic patch for non sequence-specific interactions with anionic dsDNA. A top view of the arched basic patch reveals a central channel with ∼18 Å in length and ∼14 Å in width. The channel is likely too narrow to accommodate a B-type dsDNA substrate. We propose that dsDNA associates with the arched basic patch along its border (i.e., from Lys27 to Lys60), which is ∼29 Å in length and long enough to make contact with 6–7 base pairs (Figures 3D and 3E). Accordingly, association with the arched basic patch may bend dsDNA, resulting into distortion and/or flipping of the base pairs. This hypothesis is consistent with previous reports that the (HhH)_2_ domains in other DNA metabolic enzymes are responsible for DNA bending and base pair distortion or flipping [28]–[30].

### Spatial arrangement of the L1 motif and the NTD in the *3_1_* overwound right-handed helical filament

**Figure 4 pone-0000858-g004:**
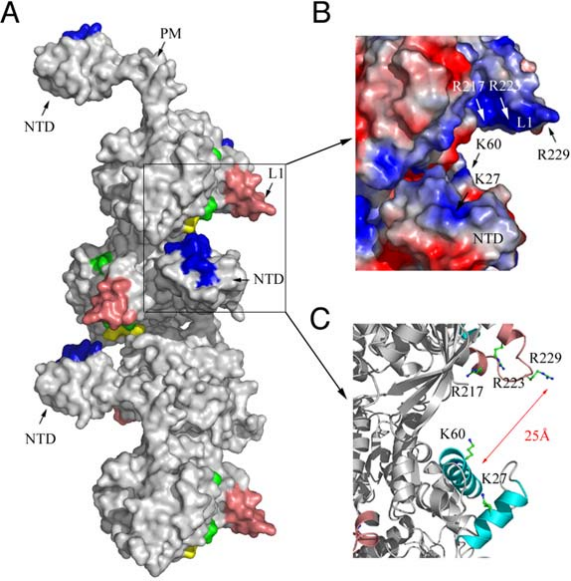
Spatial arrangement of the L1 motif and the NTD along the *3_1_* overwound right-handed *Sso*RadA filament. (A) Quaternary structure. The putative dsDNA binding regions in the NTD are shown in blue. The L1 and L2 ssDNA binding motifs are shown in pink and green, respectively. ATP binding sites are shown in yellow. The polymerization motif (PM) is indicated by an arrow. (B) A local surface charge potential of the L1 motif and the NTD region. Positive and negative charges are indicated by blue and red, respectively. (C) A ribbon diagram of two neighboring protomers (grey) showing the L1 motif (pink) and the NTD (cyan). The side chains of key basic residues K27, K60, R217, R223, and R229 are depicted in ball-and-stick representations.

L1 and NTD localize to the exterior of *3_1_* overwound right-handed helical filament (Figure 4A). A closer look at this structure revealed that the linear basic patch of the L1 motif faces the arched basic patch of the (HhH)_2_ domains at a distance of ∼25 Å (Figures 4B and 4C). Together, they constitute an outwardly open palm structure with its inner pocket containing five positively charged amino acid residues, i.e., R217, R223, R229 of the L1 motif and K27, K60 of the NTD. Intriguingly, these five basic resides are evolutionarily conserved in all arachael and eukaryotic RecA family proteins (Figures 2 and 3). It is also noteworthy that the inner pocket of this palm structure is wide enough to accommodate anionic ssDNA, dsDNA, or both via electrostatic interactions and hydrogen bonds (Figure 4A). Such an interesting structural arrangement does not exist in other crystal structures of archaeal RadA proteins, such as RecA and *Mv*RadA-AMPPNP *6_1_* right-handed filament [9], [13], [21], *Pyrococcus furiosus* (*Pf*) Rad51 ring [10], and *Sso*RadA *4_3_* left-handed helical filament [16]. One intriguing possibility is that *Sso*RadA protein in this *3_1_* overwound right-handed filament may represent or is similar to the structural intermediate or conformation during the homology pairing reaction (see Discussion).

### K27, K60, R217, R223 and R229 are all essential for *Sso*RadA promotion of D-loop formation

**Figure 5 pone-0000858-g005:**
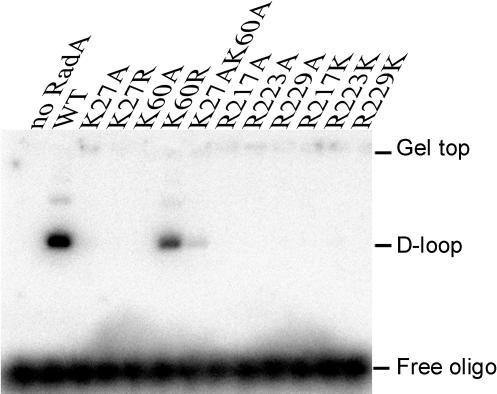
D-loop formation. *Sso*RadA-promoted homologous strand assimilation between a γ-^32^P labeled oligonucleotide and a dsDNA plasmid was carried out as described previously [16], [22]

**Figure 6 pone-0000858-g006:**
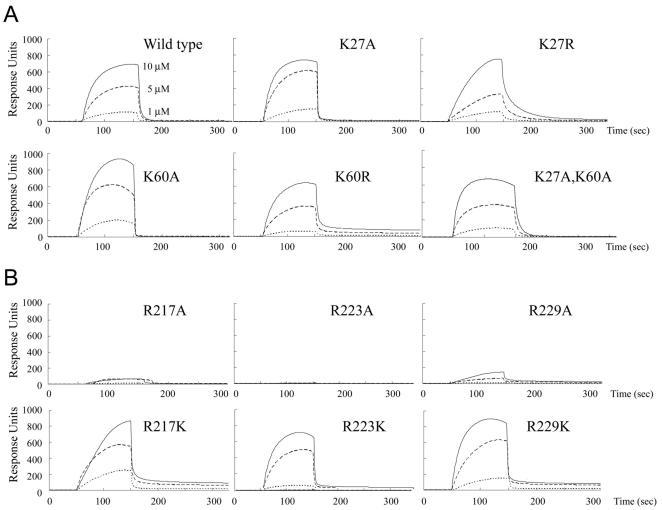
ssDNA binding. The 5′-biotinylated (dT)_50_ oligonucleotide (10 µM in nucleotides) was first injected into BIAcore SA sensor chips. Wild-type or point mutant *Sso*RadA protein (1, 5, 10 µM) was passed over the chip at 25°C. Curves represent responses with the background subtracted. Binding signal was not detected when solutions that did not contain *Sso*RadA protein were injected.

**Figure 7:**
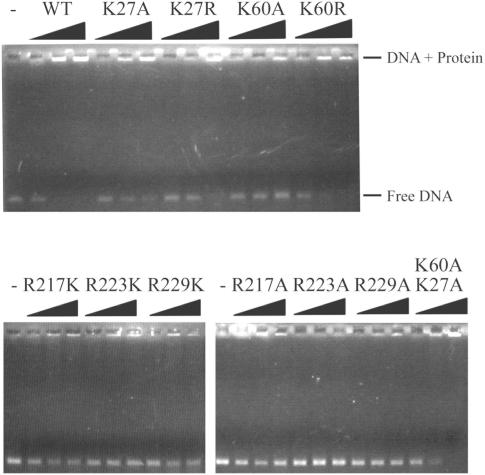
dsDNA binding. Nucleoprotein gel of reactions containing 4.3 µM (in bps) dsDNA without *Sso*RadA protein (first lane in upper panel) or with 1, 5, or 10 µM of *Sso*RadA protein. The nucleoprotein complexes were fixed with glutaraldehyde to a final concentration of 2.5%, separated from free DNA on a 0.5% agarose gel, and visualized with ethidium bromide.

To elucidate the functional roles of these five evolutionarily conserved basic residues, we first expressed and purified a panel of corresponding *Sso*RadA mutant proteins, i.e., the two lysine residues in the NTD were mutated to alanine (K27A, K60A, K27A K60A) or arginine (K27R, K60R); and the three arginine residues in the L1 motif were mutated to alanine (R217A, R223A, R229A) or lysine (R217K, R223K, R229K). We analyzed their ability to promote D-loop formation (Figure 5 and Supplementary Figure S1) and to bind ssDNA (Figure 6) and dsDNA (Figure 7). As described previously [16], [22], wild-type *Sso*RadA catalyzed homology-dependent D-loop formation between a 50 mer ^32^P-labeled oligonucleotide, P1655, and a supercoiled plasmid, GW1. We found that all mutants examined here produced no or much less D-loop product as compared to that of the wild type *Sso*RadA protein (Figure 5 and Supporting Information Figure S1). These results are in agreement with the structural results presented above, which suggest that these evolutionarily conserved lysine and arginine residues play critical roles in the catalytic function of *Sso*RadA.

### R217, R223, and R229 are indispensable for ssDNA binding

We then examined the *Sso*RadA-ssDNA interactions in real time using surface plasmon resonance (SPR) imaging by immoblizing 5′-biotinylated oligonucleotide (dT)_50_ onto the Biacore SA sensor chips. Various concentrations (1, 5, 10 µM) of the wild-type and mutant proteins were passed over the chip at 25°C in a buffer containing 2 mM MgCl_2_ and 2 mM ATP (see Material and Methods); buffers without *Sso*RadA protein were then passed over the chip for 10 min to measure association and dissociation of *Sso*RadA proteins. Binding and release of different *Sso*RadA proteins to and from ssDNA are depicted in Figure 6. Binding signals of wild-type *Sso*RadA protein gradually increased and eventually reached steady-state plateau levels (Figure 6A, upper left panel). Maximum *Sso*RadA-ssDNA binding was nearly proportional to the protein concentration. Moreover, upon washing, wild-type *Sso*RadA protein rapidly dissociated from the ssDNA substrate.

SPR analyses indicated that most mutants (except R217A, R223A and R229A) exhibited association and dissociation kinetics largely identical or similar to that of the wild-type protein. The K60A mutant had a slightly higher steady-state level, and the K27R and R217K mutants exhibited slower association rate (Figure 6A). Therefore, K27 and K60 have little or no effect on ssDNA binding. In contrast, three arginine residues (i.e., R217, R223, and R229) in the L1 motif were all indispensable for ssDNA binding. A point mutation in any of these arginines to alanine (i.e., R217A, R223A, R229A) resulted in 90–100% reduction of the SPR binding signal. On the other hand, R217K, R223K, and R229K mutants exhibited SPR binding signals similar to that of wild-type protein (Figure 6B). These results indicate that the positive charge of these three arginine residues is the main determinant for association of *Sso*RadA with ssDNA. This is compatible with the fact that *Sso*RadA and other RecA family proteins exhibit no or little nucleotide sequence specificity in binding to anionic ssDNA.

### K27 and K60 are specifically important for dsDNA binding

We also compared the ability of the *Sso*RadA protein to bind dsDNA. Wild-type *Sso*RadA protein exhibited very low SPR binding signals to a (dA-dT)_50_ dsDNA substrate (data not shown). Therefore, dsDNA binding was examined in nucleoprotein gel assays with a ∼1000 base pairs (bps) dsDNA substrate, according to a protocol used previously for yeast Rad51 protein [31]. In order to be visualized by electrophoresis on agarose gels, *Sso*RadA-dsDNA nucleoprotein complexes require stabilization by glutaraldehyde cross-linking. *Sso*RadA-dsDNA nucleoprotein complexes migrated much more slowly than free DNA. dsDNA substrate was treated with glutaraldehyde as a negative control and did not show a supershifting signal (Figure 7, the first lane of upper panel).

It is of interest that dsDNA binding to the wild-type *Sso*RadA protein saturated at ∼one protein per bp. In contrast, K60A, R223A, R229A, R223K and R229K mutants did not saturate even at ∼2.5 proteins per bp, indicating that these mutants are defective in dsDNA binding. R217A, R217K, K27A and K27R mutants also exhibited weaker affinity to dsDNA as compared to wild-type protein. In contrast, K60R mutant bound dsDNA as well as wild-type protein did (Figure 7). Taken together, these results indicate that K27, K60, R217, R223, and R229 are all important for the formation of *Sso*RadA-dsDNA complexes. Although the positive charge of R217, R223 or R229 is absolutely crucial for ssDNA binding (Figure 6), it is not the sole determinant for dsDNA binding. The results for the K60A and K60R mutants also indicate that the positive charge of K60 has a strong effect on dsDNA binding. In contrast, substitution of Lys27 with arginine could not restore the ability of *Sso*RaA to bind dsDNA. The amino group of Lys27 contacts the carbonyl group of Gly52 (Figure 3C). The equivalent residues of Lys27 and Gly52 in human Rad51 are Lys40 and Gly65. These two amino acid residues of human Rad51 had been observed to directly bind dsDNA [18]. Therefore, dsDNA is likely to make direct contacts with both Lys27 and Lys60 of *Sso*RadA protein .

It is of interest that the dsDNA binding deficiency of the K60A mutant was suppressed by an additional lysine-to-alanine substitution on K27. Still, the K27A K60A mutant is apparently defective in promoting D-loop formation (Figure 5), suggesting that binding of *Sso*RadA protein to dsDNA in the absence of ssDNA differs from that in the presence of ssDNA. Taken together, our results here support the notion that K27 and K60 have specific effects on dsDNA binding to a *Sso*RadA-ssDNA nucleoprotein helical filament (Figures 5–7).

## Discussion

Two lysines (K27, K60) in the NTD and three arginines (R217, R223, R229) in the L1 motif of *Sso*RadA are evolutionarily conserved in all archaeal and eukaryotic RecA family proteins (Figures 2 and 3). Structural and biochemical analyses in this study indicate that these five basic residues play key roles in DNA binding and strand exchange activities (Figures 2–7).

**Figure 8:**
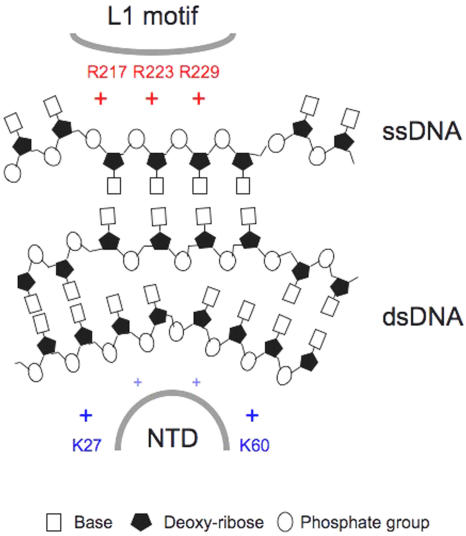
A new hypothesis for homology interactions mediated by RadA protein filaments. Interactions between three arginine residues of the L1 motif and sugar-phosphate backbone of ssDNA result in the nucleotide bases of ssDNA facing the NTD (Figure 1). An anionic dsDNA associates with the NTD along the border of a 92° basic arch via electrostatic interactions or hydrogen bonding. Lys27 and Lys60 are located at each end of this arched basic patch (Figure 3). As a result, NTD-dsDNA association may lead to DNA bending or distortion or flipping of base pairs. L1-ssDNA and NTD-dsDNA interactions function in unison to mediate homologous search and pairing between a *3_1_* overwound right-handed RadA-ssDNA nucleoprotein filament and dsDNA.

In the *3_1_* overwound right-handed helical filament of *Sso*RadA proteins, these five basic residues generate a positively charged surface for a palm structure formed by the L1 motif and the NTD. Intriguingly, this palm structure is not only localized on but also opens outwardly to the exterior of the helical filament (Figure 3). Although we can not exclude the possibility that such a structural arrangement simply arises from protein packing or filament organization during protein crystallization, we speculate that this conformation may represent or is similar to the structural intermediate responsible for homology search and pairing between ssDNA and dsDNA. Figure 8 is a cartoon that illustrates the function of the five positively charged amino acid residues. First of all, the three arginine residues in the L1 motif constitute a linear basic patch (Figure 2) for ssDNA binding via either electrostatic interactions or hydrogen bonds with the negatively charged sugar-phosphate backbone of ssDNA. This is compatible with the fact that RadA/Rad51 proteins exhibit little or no sequence specificity for DNA substrates. The ∼18 Å linear basic patch is long enough to make contact with three or four contiguous nucleotides of a ssDNA substrate, in which the purine and pyrimidine bases of bound nucleotides may be outwardly exposed. Such an arrangement not only suggests a model for RadA-mediated ssDNA stretching but also provides a framework in which a RadA-ssDNA nucleoprotein filament can form base pairs with target dsDNA (see below). Second, our structural analysis also revealed that NTD forms a 92° arched basic patch along the border of the second HhH motif (Figure 3D). Lys27 and Lys60 localize at each end of the arched basic patch and are required for dsDNA binding (Figure 7) and D-loop formation (Figure 5). The 92° arched basic patch likely associates with dsDNA along its border, and may lead to dsDNA bending or even distortion and flipping of base pairs. Taken together, we suggest that these two modes of interaction (i.e., L1-ssDNA and NTD-dsDNA) function in unison to mediate homologous pairing and strand exchange between the RadA-ssDNA nucleoprotein filament and the bound dsDNA (Figure 8).

The nature of our new model differs from that of the facilitated DNA rotation model [20], which, like other current models of RecA family proteins, is based on the assumption that these proteins are active only when they form a *6_1_* right-handed helical nucleoprotein filament. Although the latter hypothesis has been well accepted for almost two decades, it has at least two problems. First, in the *6_1_* right-handed helical filaments, all known DNA binding motifs (i.e., L1, L2, NTD, CTD) are located at or near the central axis. Therefore, the facilitated DNA rotation model assumes that a novel dsDNA binding motif(s), localized on the exterior of a *6_1_* right-handed helical nucleoprotein filament, is required for homology search, pairing, and strand exchange reactions. However, such a DNA binding motif has never been identified in any RecA family protein. In contrast, here we report that both L1 and NTD are relocated to the exterior of the *3_1_* overwound right-handed filament (Figure 4). Such a spatial arrangement allows the RadA protein to capture both ssDNA and dsDNA simultaneously. Moreover, biochemical analyses in this study have demonstrated that NTD is essential for both dsDNA binding and D-loop formation (Figures 6 and 7). Second, the facilitated DNA rotation model overlooks the fact that RecA family proteins are flexible enough to form different quaternary structures, including protein rings, *3_1_* overwound right-handed filaments and *4_3_* left-handed helical filaments. In our previous paper [16], we reported that a progressive clockwise rotation along the axes of RadA protein polymers is responsible for the structural transition from a protein ring to a *6_1_* right-handed helical filament, then to a *3_1_* overwound right-handed filament, and finally, to a *4_3_* left-handed helical filament [see Figure 5 in ref. 16]. The second and third rotation each involve a 120° discrete step. Importantly, this clockwise axial rotation accompanies the migration of L1, L2, and HhH DNA binding motifs from the interior to the exterior of RadA protein polymers; i.e., from the inner circle of a toroidal ring to the central axis of the *6_1_* right-handed filament, then to the exterior of the *3_1_* over-wound right-handed filament, and finally to the outermost surface of *4_3_* left-handed filament [see Figure 3 in ref. 16]. Thus, the L1, L2, and HhH DNA binding motifs are capable of moving simultaneously to promote ssDNA binding, homology pairing (i.e., dsDNA capturing), and finally strand exchange. We propose that DNA and RecA family protein filaments rotate simultaneously during these processes. The energy of ATP is likely used to facilitate axial rotation of RecA protein helical filaments and then to promote DNA rotation and strand exchange [16].

How is the energy of ATP hydrolysis coupled to axial rotation of RadA protein helical filaments? We had identified an arginine amino acid (Arg83) in the SRM of *Sso*RadA protein. This arginine, referred to as “R_0_”, is evolutionarily conserved in all members of RecA protein family [see Figure 1 in ref. 16]. In the RadA-AMP-PNP *6_1_* right-handed filament, the guanidinium group of R_0_ forms salt bridges with the carboxyl groups of two glutamate residues: Glu96 (denoted “E_1_”) of the same protomer and Glu157 (denoted “E_2_”) of the neighboring protomer. These two salt bridges are likely to directly control opening and closure of the ATP-binding pocket between two neighboring protomers. In the closed ring, R_0_ interacts with E_2_ but not with E_1_. In the RadA-AMP-PNP *6_1_* right-handed filament, R_0_-E_1_ and R_0_-E_2_ interactions (i.e., the “E_1_-R_0_-E_2_” triad) function as a clip to fasten the AMP-PNP or ATP binding between two protomers [see Figure 6 in ref. 16]. This model is supported by our findings that point mutations of R_0_ to glutamate in *Sso*RadA [16], *E. coli* RecA or *S. cerevisiae* Rad51 (Lin KA, Lee CD and Wang TF, unpublished results) all result in significant decreases in ATP-binding affinity. As the *6_1_* RadA helical filament undergoes clockwise axial rotation in two discrete ∼120° steps to the *3_1_* overwound right-handed filament and then to the *4_3_* left-handed filament, E_1_ and E_2_ break their ionic interactions with R_0_ and gradually move away from R_0_ [see Figure 6 in ref. 16]. Therefore, this clockwise axial rotation progressively opens up the ATP-binding pocket. We propose that the dimeric (or monomeric) conformations in the *6_1_* right-handed, *3_1_* overwound right-handed and *4_3_* left-handed filaments represent the TP (ATP-bound), DP (ADP+ Pi− or ADP-bound), and E (empty) states of RadA proteins, respectively. In this scenario, the RadA helical filament is functionally similar to the F1-ATPase. The F1-ATPase is a rotary motor in which a central gamma-subunit rotates against a surrounding cylinder made of α_3_β_3_-subunits. Driven by the three beta subunits that sequentially hydrolyze ATP, the motor also carries out “clockwise” rotation in three discrete 120° steps. These steps were denoted as TP, DP, and E [32]. Because the ATPase domains of F1-ATP and RecA family proteins are structurally and functionally conserved, we favour the possibility that the neighboring dimers or monomers along a RadA helical filament, like those of F-ATPase motor, proceed through a sequential TP-DP-E structural transition during their catalytic cycles.

One potential problem of our model arises from the stoichiometry of RadA-DNA interaction. The *3_1_* overwound right-handed filament (3 monomers per turn with 98Å pitch) may not be obtained by continuous transformation from a right-handed filament with 6 monomers per turn with 95 or 107 Å pitch, if we assume that continuous transformation requires conservation of stoichiometry (3 nucleotides or 3 base pairs per RadA). The axial spacing between consecutive bases would need to increase to at least 10.9 Å in the *3_1_* overwound right-handed filament. In fact, the actual spacing would also need to be significantly bigger than 10.9 Å since the DNA binding sites are on the exterior of the filament and the DNA would have to wrap around the filament. Such interbase distances will require breaking the DNA into mononucleotide pieces and this is certainly not what happens during DNA pairing and strand exchange. However, this dilemma may be addressed in one of two ways. First, there is no experimental evidence to support that stoichiometry (3 nucleotides or 3 base pairs per RadA) is conserved throughout the catalytic cycles of RadA or RecA family proteins. Therefore, conservation of stoichiometry may not be a prerequisite for continuous transformation. Second, it assumes that all RadA monomers in a helical filament carry out axial rotation at the same time. As described above, we speculate that RadA proteins undergo sequential structural transformation of dimeric (or monomeric) conformations from that in a *6_1_* right-handed filament (for ssDNA binding) to that in a *3_1_* overwound right-handed filament (for homology pairing), and finally to that in a *4_3_* left-handed filament (for strand exchange, ssDNA exclusion or protein dissociation) [16]. In this scenario, DNA-protein stoichiometry can be conserved and DNA substrates will also remain intact. Moreover, because the monomeric structure of RadA protein in the *3_1_* overwound right-handed filament is more extended along the axis of helical filament than those in the *6_1_* or *4_3_* filaments, it allows a transient extension or stretch of ssDNA for homology pairing (see Figure 8). Therefore, we suggest that the monomeric structure of RadA protein in the *3_1_* right-handed filament likely represents a functional form.

In summary, structural and biochemical analyses in this study and our previous paper [16] suggest a new mechanism for RadA/Rad51 mediated DNA binding, homology pairing and strand exchange reaction. We propose that RadA proteins in a helical filament sequentially carry out their catalytic function via a clockwise or right-to-left axial rotation. This is in contrast to all current models, which assume that these proteins function exclusively as 6_1_ right-handed helical filaments throughout their catalytic cycles. Ultimately, the validity of our model will have to be tested by determining the structures of RadA-DNA nucleoprotein filaments, or by visualizing ATP-fueled axial rotation of RecA family protein filaments during homology pairing and strand exchange reactions through single molecule studies.

## Materials and Methods

### Protein expression and purification

Wild-type and mutant *Sso*RadA protein were expressed and purified as described previously [16]. The amino acid sequence of purified wild-type *Sso*RadA is identical to that encoded by the *Sso*RadA gene.

### Crystallization and X-ray data collection

*Sso*RadA protein (24 mg/ml) in 30 mM Tris-HCl (pH 8.0) was crystallized using the hanging drop vapor diffusion method. Initial screening was perfomed with Hampton Research and Emerald BioStructure crystallization kits. The protein solution:reservoir ratio was 2 µl∶2 µl. Protein crystals were obtained in ∼5 days, with the reservoir containing 500 µl of 100 mM KCl, 25 mM MgCl_2_, 15% isopropanol, and 50 mM sodium cacodylate (pH 6.0). Crystals were cryoprotected by an ∼1 min wash in the reservoir solution plus 25% glycerol prior to mounting on the X-ray machine. X-ray diffraction data were collected using the National Synchrotron Radiation Research Center (NSRRC) beamline 13B1 in Taiwan (Table 1). All diffraction data were processed and scaled using the HKL2000 package [33]. The space group is *P3_1_*, with unit cell dimensions *a* = *b* = 99.55 Å, *c* = 99.41 Å and three protomers in the asymmetric unit. The three filaments along the *c* axis were related to each other by translational symmetry. Data were processed to a final resolution of 1.93 Å. The structure was determined by molecular replacement using the CNS program [34]. The twin factor was 0.5, as revealed by the twin detect function of the CNS program, indicating that it was a perfect twin structure. The search model was *Sso*RadA of the space group *P3_1_21* (PDB = 2BKE) [15]. The structure was manually rebuilt using the O program [35] and refined with the CNS program, with or without twin refinement. All figures were generated by PyMol (http://pymol.sourceforge.net). Diffraction data and refinement statistics are shown in Table 1. Surface area accessibility calculations were performed by the CCP4 program [36]. Atomic coordinates and structure factors have been deposited in the PDB under accession code 2Z43.

### DNA assimilation assays

*Sso*RadA mediated D-loop formation assays were described previously [16], [22], [37]. D-loop formation efficiency was calculated according to the molar ratio of joint molecules over total dsDNA substrate. The relative efficiency of each mutant relative to that of wild-type protein (t = 15 min) is presented.

### Surface plasmon resonance (SPR)

Interactions of wild-type and mutant *Sso*RadA protein with a single-stranded oligonucleotide (dT)_50_ were determined by a Biacore X surface plasmon resonance (SPR) biosensor instrument (Biacore at Uppsala, Sweden). The 5′-biotinylated oligonucleotide (dT)_50_ was diluted to 10 µM with HBS buffer (Biacore) and manually injected into a Biacore SA sensor chip in the channel 2 flow cell to 1300 resonance or response units. The SA sensor chip is commercially available and pre-immobilized with streptavidin (Biacore). The channel 1 flow cell was used as a reference for online background subtraction. Protein was diluted in running buffer containing HBS buffer (Biacore), 0.005% (w/v) p-20 (Biacore), 2 mM MgCl_2_, and 2 mM ATP (pH 8.0). To monitor DNA-protein interaction, *Sso*RadA protein solution (1, 5, or 10 µM in 50 µL) was injected onto the ssDNA surface with a flow rate of 30 µL/min at 25°C. The SA sensor chip was regenerated with a quick injection of 1 M NaCl and 50 mM NaOH (30 µL). Binding signals were not detected when the control buffers (i.e., no *Sso*RadA protein) were injected.

### dsDNA binding assay

We used the difference in electrophoretic mobility of glutaraldehyde-fixed *Sso*RadA-dsDNA complexes to detect the dsDNA binding ability of wild-type and mutant *Sso*RadA proteins. The experimental procedures were modified from a yeast Rad51 protocol [31]. A greater amount of glutaraldyde was applied for cross-linking, as all *Sso*RadA proteins were originally dissolved in a buffer containing 30 mM Tris-HCl (pH 8.0). An ∼1000 bp PCR product from cDNA encoding the vaccinia virus G9 protein (sequence available on request) was used as dsDNA substrate. *Sso*RadA-dsDNA complexes were formed by mixing the indicated concentrations of RadA (1, 5, or 10 µM) and dsDNA (4.3 µM in bps) in running buffer (20 mM Hepes pH 7.5, 20 mM magnesium acetate, 2 mM DTT, and 5 mM ATP) at 65°C for 30 min. Glutaraldehyde was added to a final concentration of 2.5% and the reaction incubated at 65°C for 10 min. Reaction products were loaded directly onto 0.5% agarose gels in 1X Tris-buffered EDTA, run for 70 min at 50 V (4 V/cm), and visualized with ethidium bromide.

## Supporting Information

Figure S1 (0.47 MB DOC)
