# Supplementary material for: Structural and Functional Analyses of Five Conserved Positively Charged Residues in the L1 and N-Terminal DNA Binding Motifs of Archaeal RadA Protein
Source: PLoS One. 2007 Sep 12;2(9):e858. doi: 10.1371/journal.pone.0000858 (PMC1964548; doi:10.1371/journal.pone.0000858)
Supplement: Figure S1 — (0.47 MB DOC) [file pone.0000858.s001.doc]

**Supporting Information**

**Figure S1.**

**
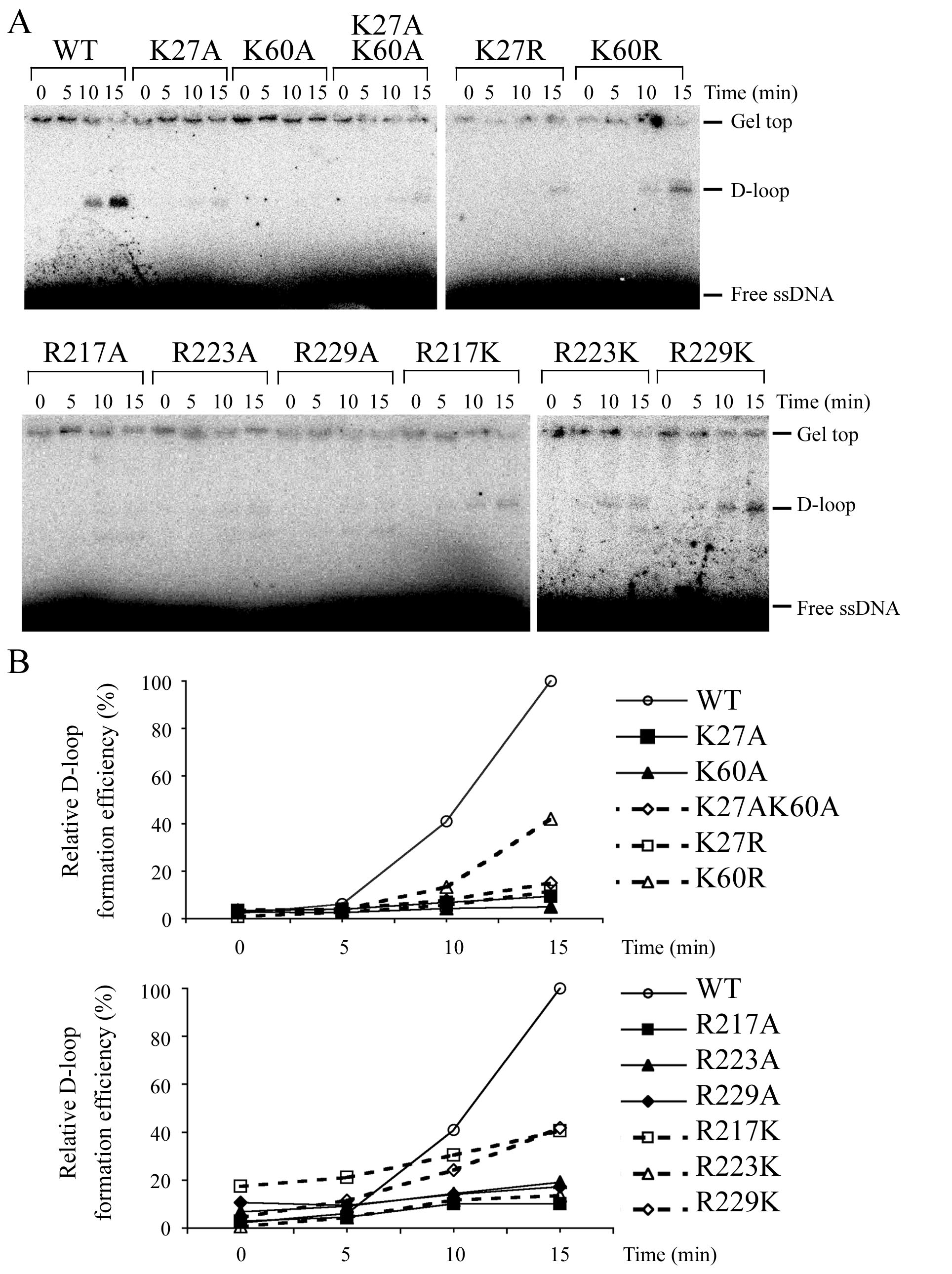
**

**Figure S1.** Time course analysis of *Sso*RadA-promoted D-loop formation. (**A**) D-loop formation assays were carried out as described previously (Lee *et al*, 2004; Chen *et al*., 2007). (**B**) Quantitation of the D-loop time course experiment shown in (**A**). The amount of D-loop is a ratio of the quantity of D-loop migrating with the GW1 plasmid DNA to the total quantity in each lane. A small fraction (~1/200) of total reaction mixture was used to determine the total radioactive count of γ−^32^P labeled oligonucleotides. The efficiency of D-loop formation was calculated according to the molar ratio of D-loop molecules over total GW1 plasmid DNA. Relative efficiency to that of wild type protein (t = 15 min) is shown.

**References:**

1. Chen LT, Ko TP, Chang YC, Lin KA, Chang CS, et al. (2007) Crystal structure of the left-handed archaeal RadA helical filament: identification of a functional motif for controlling quaternary structures and enzymatic functions of RecA family proteins. Nucleic Acids Res 35: 1787-1801.

2. Lee MH, Leng CH, Chang YC, Chou CC, et al. (2004) Self-polymerization of archaeal RadA protein into long and fine helical filaments. Biochem Biophys Res Commun 323: 845-851.
